# Genome-enabled prediction of genetic values using radial basis function neural networks

**DOI:** 10.1007/s00122-012-1868-9

**Published:** 2012-05-08

**Authors:** J. M. González-Camacho, G. de los Campos, P. Pérez, D. Gianola, J. E. Cairns, G. Mahuku, R. Babu, J. Crossa

**Affiliations:** 1 Colegio de Postgraduados, Montecillo, Edo. de México Mexico; 2 Department of Biostatistics, University of Alabama, Ryals Public Health Bldg 443, Birmingham, AL USA; 3 Department of Animal Sciences, University of Wisconsin, Madison, WI 53706 USA; 4 International Maize and Wheat Improvement Center (CIMMYT), Apdo. Postal 6-641, México DF, 06600 Mexico

## Abstract

The availability of high density panels of molecular markers has prompted the adoption of genomic selection (GS) methods in animal and plant breeding. In GS, parametric, semi-parametric and non-parametric regressions models are used for predicting quantitative traits. This article shows how to use neural networks with radial basis functions (RBFs) for prediction with dense molecular markers. We illustrate the use of the linear Bayesian LASSO regression model and of two non-linear regression models, reproducing kernel Hilbert spaces (RKHS) regression and radial basis function neural networks (RBFNN) on simulated data and real maize lines genotyped with 55,000 markers and evaluated for several trait–environment combinations. The empirical results of this study indicated that the three models showed similar overall prediction accuracy, with a slight and consistent superiority of RKHS and RBFNN over the additive Bayesian LASSO model. Results from the simulated data indicate that RKHS and RBFNN models captured epistatic effects; however, adding non-signal (redundant) predictors (interaction between markers) can adversely affect the predictive accuracy of the non-linear regression models.

## Introduction

The availability of high density panels of molecular markers has catalyzed the adoption of genomic selection (GS) methods in animal and plant breeding (Meuwissen et al. 2001); empirical evidence has demonstrated a superiority of marker-based models over pedigree-based models for predicting complex traits (e.g., VanRaden 2008; Hayes et al. 2009; de los Campos et al. 2009a; Crossa et al. 2010, 2011). However, most applications of GS use additive linear regression models, and there may be still opportunities for increasing prediction accuracy even further by capturing non-additive sources of genetic variability such as dominance or epistasis.

Evidence of epistatic effects in plant traits is vast (Holland 2001, 2006). For instance, Dudley (2008) found the presence of epistasis in oil, protein, and starch in different crosses of maize lines, and Dudley and Johnson (2010) reported that adding two locus interactions to the model increases prediction power. Despite this, experiments performed in maize have not provided sizable estimates of epistatic variance components (Hallauer and Miranda 1981), perhaps reflecting the fact that even highly epistatic systems generate a great deal of additive variance (e.g., Hill et al. 2008). Also, there is a lack of well-established methods for incorporating epistasis in the prediction of complex traits in plant breeding programs (Hallauer and Miranda 1981; Bernardo 2002).

Interactions between marker alleles at two or more loci can be accommodated in a linear model using appropriate contrasts. However, this is feasible only when the number of markers (*p*) is moderate. In GS, however, *p* is usually large, making parametric modeling of complex epistatic interactions unfeasible. An alternative is to use semi-parametric regressions (e.g., Gianola et al. 2006), such as kernel-based methods (e.g., Wahba 1978; Gianola et al. 2006; Gianola and van Kaam 2008) or neural networks (NNs) (Gianola et al. 2011), with the expectation that such procedures can capture complex higher order interaction patterns. The use of reproducing kernel Hilbert spaces (RKHS) for prediction of complex traits was first proposed by Gianola et al. (2006), and empirical studies have demonstrated good prediction accuracy in plant (e.g., Crossa et al. 2010, 2011; de los Campos et al. 2010) and chicken data (Gonzalez-Recio et al. 2008; Long et al. 2010). However, a potential limitation of RKHS regressions is that the basis functions used for regression must be defined a priori.

In NN, the basis functions are inferred from the data, giving NN great potential for capturing complex interactions between predictor variables (Hastie et al. 2009). Such flexibility comes at the price of a substantial increase in computational demands and the risk of over-fitting the training data. Radial basis function neural networks (RBFNNs) are a particular class of NN that have features that make them attractive for applications in GS. First, it has been shown that RBFNNs have universal approximation properties (e.g., Park and Sandberg 1991). Second, RBFNN combines, in a single framework, features of NNs and of RKHS, and both approaches have been widely shown to be promising for predicting phenotypes of complex traits. Further, algorithms exist [e.g., the orthogonal least-squares method proposed by Chen et al. (1991)] that make the computational burden of fitting a RBFNN much less than that of a comparable standard NN.

The RBFNNs have been applied as a prediction and classification tool in many different domains (Jayawardena and Fernando 1998; Takasaki and Kawamura 2007; Zheng et al. 2011). However, they have not been evaluated in the context of genomic selection. In this article, we (1) review the concepts of RBFNN, (2) discuss the connection between these methods and RKHS regressions, and (3) compare the predictive performance of RBFNN with that of RKHS and of an additive linear regression model (Bayesian LASSO). We also illustrate the use of these models on simulated and real maize lines genotyped with high density markers and evaluated for several trait–environment combinations.

## Materials and methods

### Simulated data sets

This data set was simulated by Zhang and Xu (2005) and has a sample size of 600 individuals. The genome has a single chromosome (1,800 cM long) and 121 evenly spaced markers with a 15 cM per marker interval. The authors simulated 9 main QTL effects and 13 interactions between different QTL effects; all QTL effects overlapped with markers. Each QTL had a contribution to phenotypic variance that varied from 0.5–20 %. Models were fitted to two simulated data sets, including the 121 evenly spaced marker covariates indicating the genotype of the *j*th marker, and the 121(121 + 1)/2 = 7,381 marker × marker first order interactions.

### Maize data sets

The maize data represent 21 trait–environment combinations measured in 300 tropical inbred lines genotyped with 55,000 SNPs each. First, we considered eight trait–environment combinations including four traits [grain yield (GY), female flowering (FFL) or days to silking, male flowering time (MFL) or days to anthesis, and anthesis-silking interval (ASI)], each evaluated under severe drought stress (SS) and in well-watered (WW) environments. This data set was previously used by Crossa et al. (2010) for the assessment of prediction performance of the BL and RKHS methods, but using only 1,148 SNPs.

Second, the 300 maize lines were evaluated in 9 international environments for gray leaf spot (GLS), a disease caused by the fungus *Cercospora zeae*-*maydis*, which is pandemic in Africa. Now recognized as one of the most significant yield-limiting diseases of maize worldwide, GLS is associated with the rapid adoption of conservation agriculture techniques. The 9 environments for GLS had appreciable levels of disease infection. Third, grain yields (GY) of these 300 maize lines were also measured in a large number of relatively high yielding environments (GY-HI) and low yielding environments (GY-LO). Finally, phenotypes for northern corn leaf blight (NCLB), a disease caused by the fungus *Exserohilum turcicum*, were taken from disease trials evaluated in two environments.

### Linear and non-linear regressions on marker genotypes

In GS, phenotypes $( {y_{i} ;\,i = 1, \ldots ,n} )$ are regressed on *p* marker covariates using a regression function that maps from marker genotypes $x_{ij} \in \{ {0,1,2} \}$ onto the real line, that is $f( {x_{i1} , \ldots ,x_{ip} } )$. Methods differ on (a) how $f( {x_{i1} , \ldots ,x_{ip} } )$ is structured (e.g., linear vs. non-linear functions of marker genotypes) and (b) how the parameters are estimated. In all models, the response variable was described as the sum of an effect common to all lines (*μ*), a genetic value *f*(**x**_*i*_), and a model residual $\bar{\varepsilon }_{i}$, that is,

$$ \bar{y}_{i} = \mu + f\left( {{\mathbf{x}}_{i} } \right) + \bar{\varepsilon }_{i} $$

Residuals were assumed to be independent draws from a normal distribution with null mean and variance equal to $\tfrac{{\sigma_{\varepsilon }^{2} }}{{n_{i} }}$, where *n*_*i*_ is defined below. Models differed in how marker information was used to describe *f*(**x**_*i*_). Phenotypes were standardized within trait-by-environment combination; therefore, in all cases the response was $\bar{y}_{i} = \frac{1}{{{\text{SD}} \times n_{i} }}\sum\nolimits_{k = 1}^{{n_{i} }} {y_{ik} }$, where *n*_*i*_ is the number of replicates available for the *i*th trait-by-environment combination, and *SD* is the sample standard deviation of the within trait-by-environment line means.

#### Linear model

In linear additive models for GS (e.g., Meuwissen et al. 2001), $f( {x_{i1} , \ldots ,x_{ip} } )$ is a weighted sum of allele dosage, that is, $f( {x_{i1} , \ldots ,x_{ip} } ) = \beta_{0} + \sum\nolimits_{j = 1}^{p} {x_{ij} \beta_{j} }$, where *β*_0_ is an intercept and $\{ {\beta_{j} } \}_{j = 1}^{p}$ are marker effects. In practice, the number of markers can vastly exceed the number of records; therefore, shrinkage estimation procedures are commonly used to estimate marker effects. This approach has been used successfully for predicting genetic values in plants and animals. However, the additive specification may not be optimal if dominance or epistasis effects make a sizeable contribution to total genetic variance. As stated, the linear additive model can be extended to accommodate dominance or epistasis by adding the appropriate effects. However, when *p* is large, modeling complex epistatic patterns using interactions is not feasible.

Here, genetic values are represented using a linear regression on marker genotypes and marker effects were estimated using the Bayesian LASSO of Park and Casella (2008), as implemented in the BLR package of R (de los Campos and Pérez 2010). Further details about this model and about the algorithms implemented in BLR can be found in de los Campos et al. (2009a) and Pérez et al. (2010). These articles also provide general guidelines for choosing hyper-parameters which were followed here to determine (1) the prior scale (*S*), (2) the degrees of freedom (*df*) of the scaled-inverse Chi-square distribution assigned to the residual variance, and (3) the shape (*s*) and rate (*r*) parameter of the gamma distribution assigned to the regularization parameter. In our implementation, *df* was set equal to 4 and the scale was set to 1, this gives a prior density with a prior expectation equal to 0.5 (i.e., one half of the sample variance of the standardized phenotypes) and it is relatively flat around its mode. Pérez et al. (2010) also provide guidelines for choosing the rate and shape parameters of the BL and the proposed approach is to choose these hyper-parameters so that the prior has a mode that is relatively flat in the neighborhood of

$$ \hat{\lambda } = \sqrt {1\frac{{\left( {1 - h^{2} } \right)}}{{h^{2} }}{{MSx}}} $$

where *MSx* is the average (across subjects) sum of squares of marker genotypes. This quantity varies across data sets. Here, we set the rate and shape parameters to 1 × 10^−4^ and 0.6, respectively; these values give a prior that has a mode close to 30 and is flat in a relatively wide range of values for $\hat{\lambda }.$

#### Reproducing kernel Hilbert spaces (RKHS) regression

The RKHS model has been suggested as an alternative to the linear model. Its proponents (e.g., Gianola et al. 2006) have argued that such a procedure may capture complex interaction patterns that may not be accounted for in the linear model, and simulations as well as empirical evidence have hinted a superiority of this approach over linear models for predicting phenotypes for some traits (e.g., de los Campos et al. 2009b, 2010; Crossa et al. 2010). In a RKHS model, the regression function takes the following form:

1 $$ f\left( {{\mathbf{x}}_{i} } \right) = \beta_{0} + \sum\limits_{{i^{\prime} = 1}}^{n} {\alpha_{{i^{\prime}}} K\left( {{\text{x}}_{i} ,{\text{x}}_{{i^{\prime}}} } \right)} $$

where ${\mathbf{x}}_{i} = ( {x_{i1} , \ldots ,x_{ip} } )^{\prime }$ and ${\mathbf{x}}_{{i^{\prime}}} = ( {x_{{i^{\prime}1}} , \ldots ,x_{{i^{\prime}p}} } )^{\prime }$ are vectors of marker genotypes, $\alpha_{{i^{\prime}}}$are regression coefficients, and $K( {{\mathbf{x}}_{i} ,{\mathbf{x}}_{{i^{\prime}}} } )$ is a positive definite function (the reproducing kernel, RK) evaluated in a pair of lines which are denoted by *i* and *i*′. This can be, for example, a Gaussian kernel, $K( {{\mathbf{x}}_{i} ,{\mathbf{x}}_{{i^{\prime}}} } ) = \exp \{ { - h\| {{\mathbf{x}}_{i} - {\mathbf{x}}_{{i^{\prime}}} } \|^{2} } \}$, where *h* is a bandwidth parameter and $\| {{\mathbf{x}}_{i} - {\mathbf{x}}_{{i^{\prime}}} } \|$ is the Euclidean distance between the vectors of marker genotypes in lines *i* and *i’*. The RK provides a set of *n* basis functions, $\{ {K( {{\mathbf{x}}_{i} , \mathbf{x}_{{i}^\prime}} )} \}_{i = 1}^{n}$, which are non-linear on marker genotypes; however, the regression function is simply a linear combination of the basis functions provided by the RK. To prevent over-fitting, the vector of regression coefficients, $( {\alpha_{1} , \ldots ,\alpha_{n} } )$, is estimated using shrinkage estimation procedures such as penalized or Bayesian regressions. Clearly, the set of basis functions is defined a priori via the choice of kernel, and an inappropriate selection may limit the ability of RKHS to capture complex patterns.

As stated above, in this model the regression function is linear on the RK. We used a Gaussian kernel, together with a strategy of kernel averaging (KA, de los Campos et al. 2010), for implicit selection of optimal values of the bandwidth parameter. In particular, we defined three extreme kernels: $K_{1} ( {{\mathbf{x}}_{i} ,{\mathbf{x}}_{{i^{\prime}}} ,h_{1} } ) = \exp ( { - \frac{{h_{1} }}{{q_{05} }} \times d_{{ii^{\prime}}}^{2} } )$, $K_{2} ( {{\mathbf{x}}_{i} ,{\mathbf{x}}_{{i^{\prime}}} ,h_{2} } ) = \exp ( { - \frac{{h_{2} }}{{q_{05} }} \times d_{{ii^{\prime}}}^{2} } )$, and $K_{3} ( {{\mathbf{x}}_{i} ,{\mathbf{x}}_{{i^{\prime}}} ,h_{3} } ) = \exp ( { - \frac{{h_{3} }}{{q_{05} }} \times d_{{ii^{\prime}}}^{2} } )$, where $d_{{ii^{\prime}}}^{2} = \sum\nolimits_{j = 1}^{p} {\frac{{( {x_{ij} - x_{{i^{\prime}j}} } )^{2} }}{{V_{j} }}}$ is a standardized squared Euclidean distance, *V*_*j*_ is the sample variance of the *j*th marker, *q*_05_ is the 5th percentile of $d_{{ii^{\prime}}}^{2}$, and $h_{1} = 5;\,h_{2} = 1;\,h_{3} = {1 \mathord{/ {\vphantom {1 5}} \kern-\nulldelimiterspace} 5}$ are values of the bandwidth parameter, such that $K_{1} ( {{\mathbf{x}}_{i} ,{\mathbf{x}}_{{i^{\prime}}} ,h_{1} } )$ gives extremely local basis functions and $K_{3} ( {{\mathbf{x}}_{i} ,{\mathbf{x}}_{{i^{\prime}}} ,h_{3} } )$ gives basis functions with a much wider span. Figure 4 (Appendix 1) gives a histogram (for the ASI-SS maize data set) of the off-diagonal entries of the three kernels. *K*_1_ has very small off-diagonal values, *K*_2_ gives off-diagonal values concentrated in the [0.2, 0.6] interval and *K*_3_ gives off-diagonal values concentrated in the [0.7, 0.9] interval.

Kernel averaging was implemented using Bayesian methods, as described by de los Campos et al. (2010). The joint prior distribution of this Bayesian RKHS regression has eight hyper-parameters; the prior scale (*S*) and degrees of freedom (*df*) of the scaled-inverse Chi-square distribution assigned to the residual variance, and those of the distributions assigned to the variances associated with each of the three RK (the scale and the degrees of freedom hyper-parameters). In our implementation, we set the *df* = 4, because this gives relatively un-informative priors, and chose the scale parameters so that (1) the prior expectation of the residual variance was one half of the sample variance of the standardized phenotypes (in our case ${\text{S}} = {{( {df - 2} )} \mathord{/ {\vphantom {{( {df - 2} )} 2}} \kern-\nulldelimiterspace} 2} = 1$) and (2) the prior expectation of the variance of each of the kernels was 1/6 of the sample variance of standardized phenotypes (in our case ${\text{S}} = {{( {df - 2} )} \mathord{/ {\vphantom {{( {df - 2} )} 6}}  \kern-\nulldelimiterspace} 6} = {1 \mathord{/ {\vphantom {1 3}} \kern-\nulldelimiterspace} 3}$).

#### Single hidden layer neural network

In a NN, the basis functions are inferred from the data, which give NN great flexibility in terms of capturing complex patterns. The rest of this section gives an overview of these procedures. We begin by reviewing a standard single hidden layer NN for a continuous response with the RBFNN introduced subsequently.

**Fig. 1:**
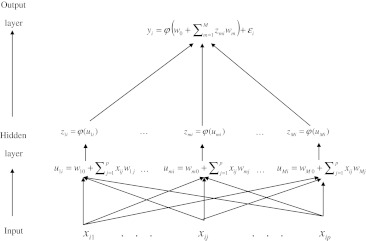
Graphical representation of a single hidden layer feed-forward neural network (NN). In the hidden layer, input variables ${\mathbf{x}}_{i} = ( {x_{i1} , \ldots ,x_{ip} } )$ ($j = 1, \ldots , p$ markers) are combined using a linear function, $u_{mi} = w_{m0} + \sum\nolimits_{j = 1}^{p} {x_{ij} w_{mj} }$ (*m* = 1,…,*M*), and subsequently transformed using a non-linear activation function, $\varphi_{m} ( \cdot )$, yielding a set of *M* (*M* = number of neurons) inferred scores, $\, z_{mi} = \varphi_{m} ( {u_{mi} } )$. These scores are used in the output layer as basis functions to regress the response using the linear activation function on the data-derived predictors $y_{i} = \varphi ( {w_{0} + \sum\nolimits_{m = 1}^{M} {z_{mi} w_{m} } } ) + \varepsilon_{i}$; $\varphi ( \cdot )$ could be either an identity or any other function

A graphical representation of a single hidden layer neural network is given in Fig. 1. This NN can be thought of as a two-stage regression (e.g., Hastie et al. 2009). In the first stage (hidden layer), *M* data-derived basis functions, $\{ {z_{mi} } \}_{i = 1;m = 1}^{i = n;m = M}$, are inferred; in the second stage (the output layer), the response is regressed on the basis functions (inferred in the hidden layer) using a non-linear procedure (Fig. 1).

In the hidden layer, one data-derived predictor (or basis function) is inferred at each of *M* neurons. These data-derived predictors are formed by first inferring a score (*u*_*mi*_), which is a linear combination of the input variables (marker genotypes, in our case), and then transforming this score using a non-linear activation function, $\varphi ( \cdot )$, that is $z_{mi} = \varphi_{m} ( {u_{mi} } ) = \varphi_{m} ( {w_{m0} + \sum\nolimits_{j = 1}^{p} {x_{ij} w_{mj} } } )$, where *w*_m0_ is an intercept (also referred to as ‘bias’ term), and ${\mathbf{w}}_{m} = \{ {w_{mj} } \}_{m = 1;j = 1}^{m = M;j = p}$ is a vector of regression coefficients (the so-called ‘weights’).

Subsequently, in the output layer, phenotypes are regressed on the data-derived features, $\{ {z_{mi} } \}_{i = 1;m = 1}^{i = n;m = M}$, according to $y_{i} = \varphi ( {w_{0} + \sum\nolimits_{m = 1}^{M} {z_{mi} w_{m} } } ) + \varepsilon_{i}$, where $\varphi ( \cdot )$ is usually a linear activation function and *ɛ*_*i*_ is a model residual. For a continuous outcome, $\varphi ( \cdot )$, may simply be an identity link, so that $y_{i} = w_{0} + \sum\nolimits_{m = 1}^{M} {z_{mi} w_{m} } + \varepsilon_{i}$. Model specification in NN refers to the choice of architecture (i.e., the number of hidden layers and of neurons per hidden layer) and the type of activation function.

The activation function is a monotonic map from a score defined in the real line onto the interval [0, 1] (for a sigmoid function) or onto the interval [-1, 1] (for a hyperbolic tangent function). For example, the sigmoid function is $z_{mi} = \varphi_{m} ( {u_{mi} ,\theta } ) = \frac{1}{{1 + \exp ( { - \theta \,u_{mi} } )}}$, where *θ* is a parameter controlling the shape of the activation function. The use of data-derived predictors and activation functions, together with the possibility of using multiple neurons and layers, gives NN great flexibility for capturing complex interaction patterns between predictors; however, the computational burden can be extremely high and over-fitting may occur.

#### Radial basis function neural network

**Fig. 2:**
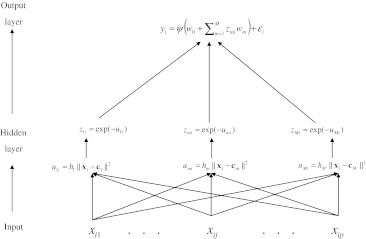
Graphical representation of a single hidden layer (Gaussian) radial basis function neural network (RBFNN). In the hidden layer, information from input variables $(x_{i1} , \ldots ,x_{ip} )$ ($j = \,1, \ldots ,p$ markers) is first summarized by means of the Euclidean distance between each of the input vectors {**x**_*i*_} with respect to *M* (data-inferred) (*M* = number of neurons) centers {**c**_*m*_}, that is $u_{mi} = h_{m} ||{\mathbf{x}}_{i} - {\mathbf{c}}_{m} ||^{2}$. These distances are then transformed using the Gaussian function, $z_{mi} = \exp ( - u_{mi}^{{}} )$, yielding *M* data-derived scores. These scores are used in the output layer as basis functions for the linear regression, $y_{i} = \psi ( {w_{0} + \sum\nolimits_{m = 1}^{M} {z_{mi} w_{m} } } ) + \,\varepsilon_{i}$; $\psi ( \cdot )$ is usually an identity function

The RBFNN was first proposed by Broomhead and Lowe (1988) and Poggio and Girosi (1990), who applied regularization theory to solve ill-conditioned problems in the approximation/interpolation of a function. Figure 2 gives a graphical display of a single hidden layer RBFNN with *M* neurons (*M* ≤ *n*). The output layer is exactly as that shown for NN in Fig. 1; the main difference between the standard NN and an RBFNN is how the hidden layer is structured, that is, how the basis functions are inferred. In an RBFNN, the basis functions consist of a pre-determined number of radial basis functions (RBFs), each of which is indexed by parameters (e.g., centroid; see below for further explanation) to be estimated from the data.

A radial basis function, $\psi ( \cdot )$, is a map of pairs of vectors, $\{ {{\mathbf{x}}_{i} ,{\mathbf{c}}} \}$, onto the real line, with the peculiarity that the map depends only on the Euclidean distance between the two vectors (input vector, **x**_*i*_, and centroid vector, **c**), that is, $\psi ( {{\mathbf{x}}_{i} ,{\text{c}}} ) = \psi ( {\| {{\mathbf{x}}_{i} - {\mathbf{c}}} \|} )$. The Gaussian kernel is a particular case of this. The illustration in Fig. 2 uses a Gaussian RBF; however, the methodology can be applied using other RBFs, such as splines, multi-quadrics, etc. For a given set of centroids $\{ {{\mathbf{c}}_{1} , \ldots ,{\mathbf{c}}_{M} } \}$ (*M* vectors each of order *p*), the set of parameters involved in an RBFNN (the weights of the output layer, ${\varvec{\omega}} = \{ {w_{0} ,.w_{1} .,w_{M} } \}$) can include a large number of unknowns; thus, shrinkage estimation methods may be needed. The regularization approach for solving a learning (approximation/interpolation) problem is to search for a function $f( {{\mathbf{x}}_{i} ,{\varvec{\omega}}} )$ that approximates the training set of response data; this function has input vectors, ${\mathbf{x}}_{i} \, \in \,R^{p}$ (the domain of the function), responses $y_{i} \, \in \,R,\;( {i = 1, \ldots ,n} )$, and the weight vector ***ω***. In other words, we need to find the functional $\Upphi [ {f( {{\mathbf{x}}_{i} ,{\varvec{\omega}}} )} ]$ that minimizes the cost function $H[ {f( {{\mathbf{x}}_{i} ,{\varvec{\omega}}} )} ]$ (Poggio and Girosi 1990; Kecman 2001),

$$ H\left[ {f\left( {{\mathbf{x}}_{i} ,{\varvec{\omega}}} \right)} \right]\mathop = \limits_{\begin{subarray}{l} \arg \min \\ \,\,\,\,\,\,\omega \end{subarray} } \,\sum\limits_{i = 1}^{n} {\left( {y_{i} - f\left( {{\mathbf{x}}_{i} ,{\varvec{\omega}}} \right)} \right)^{2} } + \lambda \Upphi \left[ {f\left( {{\mathbf{x}}_{i} ,{\varvec{\omega}}} \right)} \right] $$

where $\sum\nolimits_{i = 1}^{n} {( {y_{i} - f( {{\mathbf{x}}_{i} ,{\varvec{\omega}}} )} )^{2} }$ is a residual sum of squares between the response *y*_*i*_ and the approximating function $f( {{\mathbf{x}}_{i} ,{\varvec{\omega}}} )$ (i.e., a measure of goodness-of-fit); *λ* is a small, positive number (the Lagrange multiplier), also called the regularization parameter, that controls the trade-off between fitness and model complexity; $\Upphi [ {f( {{\mathbf{x}}_{i} ,{\varvec{\omega}}} )} ]$ is a measure of complexity of $f( {{\mathbf{x}}_{i} ,{\varvec{\omega}}} )$ and a penalty function also called a stabilizer that enforces the smoothness of $f( {{\mathbf{x}}_{i} ,{\varvec{\omega}}} )$. The regularization parameter *λ*, which is commonly proportional to the extent of noise in data, determines the influence of this stabilizer and controls the trade-off between the two terms of $H[ {f( {{\mathbf{x}}_{i} ,{\varvec{\omega}}} )} ]$. The stabilizer (or penalty) function $\Upphi [ {f( {{\mathbf{x}}_{i} ,{\varvec{\omega}}} )} ]$ can take several forms (i.e., spline, multi-quadric, radial basis, Gaussian, etc.).

When $\Upphi [ {f( {{\mathbf{x}}_{i} ,{\varvec{\omega}}} )} ]$ takes a symmetrical radial form, a particular regularized solution that minimizes $H[ {f( {{\mathbf{x}}_{i} ,{\varvec{\omega}}} )} ]$ is given by the linear combination of the Gaussian RBFs (Poggio and Girosi 1990; Kecman 2001):

$$ f\left( {{\mathbf{x}}_{i} ,{\varvec{\omega}}} \right)\, = \,w_{0} + \sum\limits_{m = 1}^{M} {w_{m} \psi_{m} \left( {\left\| {{\mathbf{x}}_{i} - {\mathbf{c}}_{m} } \right\|} \right)} $$

where *w*_0_ is the intercept, *w*_*m*_ are the weights of the linear layer, **c**_*m*_ are the centers of the RBFs and $\psi_{m} ( {\| {{\mathbf{x}}_{i} \, - \,{\mathbf{c}}} \|} ) = \exp [{ - h\| {{\mathbf{x}}_{i} \, - \,{\mathbf{c}}_{m} } \|^{2} }]$ are Gaussian RBFs that depend only on the Euclidean norm of the difference vector ${\mathbf{x}}_{i} - {\mathbf{c}}_{m}$. The weights (*w*_*m*_), the centroids (**c**_*m*_), and *h* are estimated in such a way that the fit between $f( {{\mathbf{x}}_{i} ,{\varvec{\omega}}} )$ and the desired response is optimum.

#### Estimating the parameters of the RBFNN

To estimate the parameters of a RBFNN, the weights *w*_*m*_ of the linear output layer are determined using the ordinary least-squares method, once the RBF (Gaussian in this case) $\psi_{m} ( \cdot )$ (0 < *m* ≤ *M*), their corresponding centers, and the bandwidth *h* of the RBF have been assigned values. Several methods are available for selecting the centers (Haykin 1994); in this study, the centroids were selected using the orthogonalization least-squares procedure proposed by Chen et al. (1991). This method sequentially selects the centers of the RBF such that each new selected center is orthogonal to the previous ones. The selected centers maximize the decrease in the mean squared error of the RBFNN, and the algorithm stops when the number of centers attains a desired precision, or when the number of centers is equal to the number of input vectors, that is, when *M* = *n*.

### Relationship between RBFNN and RKHS

The RBFNN is closely related to RKHS regression. In particular, if in Fig. 2 we let the activation function of the output layer be the identity function $\psi ( {w_{0} + \sum\nolimits_{m = 1}^{M} {z_{mi} w_{m} } } ) = w_{0} + \sum\nolimits_{m = 1}^{M} {z_{mi} w_{m} }$ and the number of neurons be equal to *n*, with ${\mathbf{c}}_{m} = {\mathbf{x}}_{{i^{\prime}}}$, then the structure of the conditional expectation function of the RKHS regression and the structure of the RBFNNs are exactly the same. In the RBFNN, the strategy is to select a set of basis functions by estimating centers (**c**_*m*_), and each center defines a basis function. Typically, the number of centers (or neurons, in this case) is much smaller than the number of data points. The strategy in RKHS regression is different: a large set of basis functions is offered to the algorithm (at least *n*, one per data point, and more, when kernel averaging is used; see Eq. [1]), but the contribution of each of these basis functions to the conditional expectation (i.e., the *α*’s) is estimated using shrinkage estimation procedures. In the statistical learning literature, this is known as ‘automatic knot selection’ (Ruppert et al. 2003) and is the strategy used by the smoothing spline (Wahba 1990). Arguably, the performance of an RBFNN could be improved if a shrinkage estimation procedure was used, instead of the least-squares method of Chen et al. (1991), but the latter is computationally simpler.

### Model comparison

The predictive ability of the additive Bayesian LASSO linear model, RKHS, and the RBFNN was evaluated. A total of 50 independent random partitions of each of the 23 data sets into training (90 % of the data points) and testing (10 % of the data points) were generated. For each of these partitions, models were fitted to the training set data, and prediction accuracy was evaluated in the testing data set. Accuracy was assessed by means of Pearson’s correlation between predictions and observations and by the predictive mean squared error (${\text{PMSE}} = n_{tst}^{ - 1} \sum\nolimits_{i = 1}^{{n_{test} }} {(\bar{y}_{i} - \hat{\bar{y}}_{i} )}^{2}$, where $\hat{\bar{y}}_{i}$ is the predicted value), both evaluated in testing data sets of size *n*_*tst*_.

The number of times a given model had a higher correlation (or smaller PMSE) than another was counted and represented in a graph, to produce a visual assessment of the “winner” models in terms of correlation and PMSE.

## Results

The average (across 50 training–testing partitions) correlations between predictions and observations obtained with the simulated and real data sets are given in Table 1. Results for PMSE are given in Table 2 (Appendix 2). Given the similarity of results for correlations and PMSE, here we will concentrate on correlations only.

### Simulated data sets

The analysis involving 121 marker covariates showed a marked superiority of RKHS (correlation 0.757) and of RBFNN (correlation 0.770) over the Bayesian LASSO (correlation 0.643). Here, RBFNN outperformed RKHS slightly. These results confirm that RKHS and RBFNN are able to capture patterns (perhaps generated by epistatic effects) that cannot be detected by a linear model for additive effects.

However, when marker main effects and two-marker interactions were fitted, the performance of the linear model increased markedly (correlation 0.797) and that of the semi-parametric procedures decreased (average correlation 0.547, for both RKHS and RBFNN). These results indicate that, for non-linear models, the information on interaction between predictors incorporated into the input space becomes redundant in the feature space. On the other hand, for the linear model, the information on the marker × marker interaction incorporated in the input space is useful to predict the feature space. The linear model was able to detect this via estimates of regression coefficients which weight the contribution of each marker to the estimated conditional expectation. On the other hand, in both RBFNN and RKHS, each marker gets a similar weight in the basis function or kernel, and the effect of adding non-signal covariates reduces method performance.

### Maize data sets

Overall, that is, averaged across training–testing partitions, the three methods performed similarly, with only a slight superiority of RKHS (average correlation 0.553) over RBFNN (average correlation 0.547) and the linear model (average correlation 0.542) (Table 1). Similarly, RKHS had a slightly smaller average PMSE (0.645) than RBFNN (0.656) and BL (0.658) (Table 2).

**Table 1:** Mean correlation of three models, Bayesian LASSO (BL), reproducing kernel Hilbert space (RKHS) regression, and radial basis function neural network (RBFNN), and the number of times one model has a higher correlation than the other (RKHS > BL, RBFNN > BL, and RKHS > RBFNN) for 50 random partitions for each of 23 individual data sets (trait–environment combinations) and across 21 maize data sets

Trait–environment	BL	Mean correlation		Number of times a model is better than the other		
		RKHS	RBFNN	RKHS > BL	RBFNN > BL	RKHS > RBFNN
Simulated data sets						
121 Markers	0.643	0.757	0.770	50	50	5
7,381 Markers	0.797	0.547	0.547	0	0	26
Maize data sets						
FFL-WW	0.814	0.836	0.834	37	32	34
FFL-SS	0.754	0.763	0.757	30	32	22
MFL-WW	0.817	0.841	0.832	37	32	36
MFL-SS	0.776	0.782	0.780	31	36	27
ASI-WW	0.582	0.586	0.594	27	32	23
ASI-SS	0.612	0.621	0.605	34	23	31
GY-SS	0.326	0.330	0.288	28	13	36
GY-WW	0.557	0.548	0.529	16	13	33
GY-HI	0.633	0.663	0.653	37	37	24
GY-LOW	0.410	0.402	0.393	37	31	30
GLS 1	0.220	0.259	0.260	12	20	21
GLS 2	0.419	0.439	0.431	36	17	35
GLS 3	0.590	0.579	0.582	23	25	22
GLS 4	0.522	0.544	0.506	20	24	20
GLS 5	0.346	0.332	0.344	39	38	23
GLS 6	0.284	0.263	0.278	9	25	18
GLS 7	0.477	0.502	0.508	36	16	38
GLS 8	0.596	0.584	0.592	42	29	31
GLS 9	0.522	0.544	0.506	24	21	26
NCBL 1	0.644	0.709	0.691	49	45	40
NCBL 2	0.478	0.491	0.525	34	36	15
Combined 21 maize trait–environments						
	0.542	0.553	0.547	688	627	616

*FFL* female flowering, *MFL* male flowering, *ASI* MFL to FFL interval, *GY* grain yield, *SS* severe drought stress, *WW* well-watered environment, *HI* optimum environment, *LOW* stress environment, *GLS Cercospora zeae*-*maydis*, *NCLB* *Exserohilum turcicum*

**Fig. 3:**
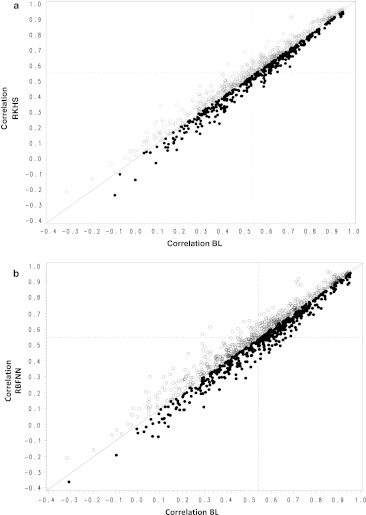
Plot of the correlation for each of 50 partitions in each of 21 trait–environment combinations for different combinations of models. In **a** when the best model is RKHS, this is represented by a *white circle*; when the best model is BL, this is represented by a *black circle*. In **b** when best model is RBFNN, this is represented by a *white circle*; when the best model is BL, this is represented by a *black circle*

**Fig. 4:**
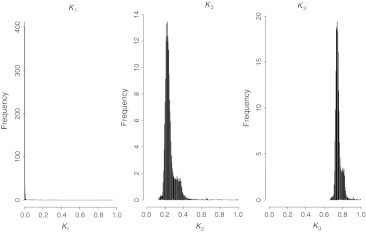
*Histograms* of the off-diagonal entries of each of the three kernels used (*K*_1_, *K*_2_, *K*_3_) in the RKHS model for the ASI-SS maize data set

**Fig. 5:**
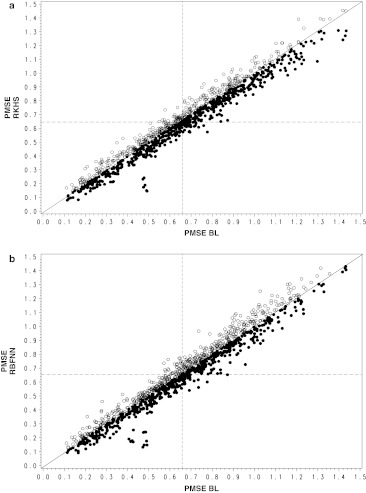
Plot of the PMSE for each of the 50 partitions in each of 21 trait–environment combinations for different combinations of models. In **a** when the best model is RKHS, this is represented by a *white circle*; when the best model is BL, this is represented by a *black circle*. In **b** when best model is RBFNN, this is represented by a *white circle*; when the best model is BL, this is represented by a *black circle*

Figure 3a–b (and Fig. 5a–b in Appendix 2) gives the correlations (and PMSE) obtained with RKHS versus BL, and RBFNN versus BL. In these figures, each dot represents the estimated correlations (and PMSE) for each of the two methods included in the plot and corresponds to one of the 1,050 analyses (21 trait–environment combinations × 50 training–testing partitions) conducted. A point above the 45° line represents an analysis where the method whose predictive correlation (and PMSE) is given on the vertical axis outperformed the one whose correlation (and PMSE) is given on the horizontal axis. Although there is a slight overall superiority of RKHS and RBFNN over the linear model (they outperformed the linear model 66 and 60 % of the times, respectively; see Table 1), the average performance across traits and environments was similar.

#### Flowering (FFM, MFL, ASI)

For traits FFL and MFL (Table 1), the three models achieved high prediction accuracy (correlations over 0.75), whereas for ASI they achieved moderate correlations. These results are in agreement with those reported by Crossa et al. (2010) for these traits. For FFL and MFL, the predictive accuracy obtained under well-watered conditions was higher and more stable (across partitions) than that obtained under drought stress. For these traits, we observed, in general, a slight superiority (1–3 % in the correlation) of RKHS or RBFNN over the additive Bayesian LASSO.

#### Grain yield

For yield traits (Table 1) we obtained moderately high correlations in well-watered (GY-WW) and high-yield environments (GY-HI), and a lower predictive performance under drought stress (GY-SS) and low-yield environments (GY-LO). These results highlight the difficulties of predicting performance under stress conditions and reinforce the importance of having a precise phenotypic system for controlling local plot-to-plot variability in field trials under restrictive conditions. The analysis of GY traits showed slightly better prediction of BL and RKHS over RBFNN.

#### Gray leaf spot

Estimated predictive correlations ranged from 0.220 to 0.596, depending mostly on environment. Although there were some differences across models, their ranking was not clear; the BL, RKHS and RBFNN methods were best in 4, 3 and 2 of the 9 environments, respectively.

#### Northern corn leaf blight

The estimated predictive correlations for these trait–environment combinations were moderate to high, and in the two environments we observed better performance of the semi-parametric procedures: RKHS was best in environment 1 and RBFNN was best in environment 2.

## Discussion and conclusions

Our empirical results, in which 21 maize data sets represented different traits and environments, indicated that the three models considered had a very similar overall prediction accuracy, with a slight superiority of RKHS and RBFNN over the additive Bayesian LASSO model. In general, these results are similar and sometimes slightly better than other findings using similar data sets. The sample size (300 maize lines) may be a limiting factor for obtaining better discrimination between the predictive accuracy of these models. Results from the simulated data suggest that, for non-linear models, introducing interactions between predictors (markers) in the input space may not be necessary for predicting the feature space; however, this interaction information in the input space is necessary (but feasible to be incorporated in real situations) when the feature space is predicted by means of a linear model. These results were confirmed when using real data.

The simulated data example not only shows that RKHS or RBFNN can capture epistatic patterns, but also indicates that adding non-signal predictors (as might happen using 55  K, 100 K or denser platforms) can adversely affect the predictive accuracy of these models, because in the current formulations of RKHS and RBFNN all markers are equally weighted. Possible ways to overcome this problem would be to (1) introduce unknown marker weights in the kernel, which could be computationally challenging; (2) use arbitrary weights or pre-selecting markers based on an ad-hoc procedure (e.g., single marker regression or information gain); or (3) obtain haplotypes and examine their prediction accuracy. This is an issue that requires further study.

Given the hundreds of thousands of markers, including all pair-wise (or higher order) interactions among markers in linear models becomes a difficult and almost impossible problem to solve. As pointed out initially by Gianola et al. (2006), and subsequently corroborated by Long et al. (2010), non-parametric models do not impose strong assumptions on the phenotype–genotype relationship and allow capturing interactions among loci. The results of these real data sets, comprising maize trials conducted to measure several traits under a wide range of environmental conditions, agreed with previous findings in animal breeding and with simulated experiments in the sense that sometimes a non-parametric treatment of markers may account for epistatic effects that are not captured by linear additive regression models.

The two kernel models considered, RBFNN and RKHS, had some similarities and displayed good predictive abilities in several trait–environment combinations. While RKHS with kernel averaging is robust for any combination of traits and environments, the two non-parametric models, RBFNN and RKHS, seem to be useful for predicting quantitative traits with complex underlying gene action under varying types of interaction with different environmental conditions. While the additive linear model seems to be robust when hundreds of non-signal predictors are included in the model, the degraded performance of RKHS and RBFNN when a large number of non-signal markers are added to the model requires further investigation, along the previously described lines.

Although parametrically estimating all possible regression coefficients in a linear model is not feasible for large *p*, it is possible to make further improvements on the accuracy of the RKHS and RBFNN models by introducing differential weights in SNPs, as shown by Long et al. (2011) for RBFs. Further, the output layer of the RBFNN used in this study does not use a regularized regression but, rather, ordinary least squares. Using a shrinkage regression model for the output layer of the RBFNN may offer an extra increase in accuracy. This needs further investigation in the context of genomic prediction.

## Appendix 2

**Table 2:** Mean predicted mean squared error (PMSE) of three models, Bayesian LASSO (BL), reproducing kernel Hilbert space (RKHS) regression, and radial basis function neural network (RBFNN), and the number of times one model has a higher correlation than the other (RKHS > BL, RBFNN > BL, and RKHS > RBFNN) for 50 random partitions for each of 23 individual data sets (trait–environment combination) and across 21 maize data sets

Trait–environment	BL	Mean PMSE		Number of times a model is better than the other		
		RKHS	RBFNN	RKHS > BL	RBFNN > BL	RKHS > RBFNN
Simulated data sets						
121 Markers	0.583	0.431	0.404	50	50	4
7,381 Markers	0.371	0.699	0.693	0	0	19
Maize data sets						
FFL-WW	0.249	0.219	0.202	34	34	23
FFL-SS	0.342	0.330	0.337	31	28	28
MFL-WW	0.251	0.221	0.220	32	31	32
MFL-SS	0.319	0.311	0.311	28	27	27
ASI-WW	0.649	0.650	0.642	24	31	20
ASI-SS	0.654	0.649	0.670	27	21	27
GY-SS	0.888	0.888	0.927	29	11	32
GY-WW	0.675	0.693	0.706	16	14	29
GY-HI	0.595	0.571	0.575	36	26	27
GY-LOW	0.843	0.855	0.874	34	29	30
GLS 1	0.957	0.918	0.959	13	19	24
GLS 2	0.832	0.815	0.824	30	15	37
GLS 3	0.621	0.635	0.632	26	20	29
GLS 4	0.730	0.713	0.751	25	20	29
GLS 5	0.819	0.817	0.837	37	36	21
GLS 6	0.969	0.971	0.994	13	25	22
GLS 7	0.756	0.729	0.724	26	15	35
GLS 8	0.616	0.630	0.621	37	32	27
GLS 9	0.732	0.712	0.750	23	16	32
NCBL 1	0.595	0.519	0.534	49	47	32
NCBL 2	0.724	0.708	0.68	35	40	15
Combined 21 maize trait–environments						
	0.658	0.645	0.656	655	587	601

*FFL* female flowering, *MFL* male flowering, *ASI* MFL to FFL interval, *GY* grain yield, *SS* severe drought stress, *WW* well-watered environment, *HI* optimum environment, *LOW* stress environment, *GLS Cercospora zeae*-*maydis*, *NCLB Exserohilum turcicum*
